# Passive head-up tilt positioning as an early mobilization strategy in neurocritical care: a prospective-retrospective controlled study

**DOI:** 10.3389/fneur.2025.1615514

**Published:** 2025-08-08

**Authors:** Geng Jia, Yi Feng, Zhenwei Liu, Changchun Yang, Ya Peng, Naiyuan Shao

**Affiliations:** Department of Neurosurgery, First People's Hospital of Changzhou, Changzhou, China

**Keywords:** neurocritical care, early mobilization, passive head-up tilt positioning, intracranial pressure, delirium, mechanical ventilation, functional recovery, tilt table therapy

## Abstract

**Background:**

Early mobilization is recommended in neurocritical care, yet passive mobilization strategies for patients with impaired consciousness remain underexplored. This retrospective matched cohort study, incorporating prospectively collected intervention data, evaluates the clinical efficacy and physiological impact of passive head-up tilt positioning in patients with severe neurological injury.

**Methods:**

We conducted a prospective-retrospective matched cohort study involving 58 patients with traumatic brain injury or hypertensive intracerebral hemorrhage. Twenty-nine patients received standardized passive verticalization using a motorized standing bed; 29 matched controls received standard care. Intracranial dynamics (ICP, CPP, and PRx), respiratory mechanics, intra-abdominal pressure (IAP), and neurological assessments (GCS, CRS-R, ICDSC) were measured at defined intervals. Primary outcomes included NSICU/hospital length of stay, duration of ventilation, complication rates, and long-term functional outcomes.

**Results:**

Passive verticalization was well-tolerated and associated with a significant reduction in ICP (10.62 ± 2.13 vs. 8.38 ± 2.27 mmHg, *p* < 0.05) without affecting CPP or PRx. Neurological function improved significantly (GCS: 7.90 → 10.07; CRS-R: 8.17 → 12.03; all *p* < 0.05), and delirium severity declined (ICDSC: 5.97 → 1.62). Intervention patients had shorter NSICU and hospital stays, reduced mechanical ventilation duration, earlier enteral nutrition, lower DVT incidence, and superior 6-month ADL and DRS scores.

**Conclusion:**

Passive head-up tilt positioning is a safe, feasible early mobilization strategy in neurocritical care. It improves neurological recovery, reduces complications, and supports long-term functional outcomes. These findings support the incorporation of passive verticalization into early rehabilitation protocols for patients unable to engage in active mobilization.

## Introduction

Early mobilization is widely recognized as a fundamental aspect of critical care rehabilitation, strongly advocated by international guidelines for general intensive care unit (ICU) patients (1–3). Multiple studies have demonstrated its efficacy in reducing complications, shortening both ICU and overall hospital lengths of stay (LOS), enhancing functional recovery, and increasing patient satisfaction (4–8). For neurocritical care patients specifically, early mobilization holds particular importance due to their elevated risk of prolonged immobilization complications, impaired consciousness recovery, and neurological deficits, underscoring the need for tailored mobilization strategies to facilitate neurological improvement and reduce secondary complications. However, evidence specifically addressing the benefits of early mobilization in neurocritical care patients remains scarce and inconsistent (9–13). Patients in neurocritical care settings frequently present with impaired consciousness, which limits patient cooperation; heavy sedation and paralysis, reducing voluntary movement; mechanical ventilation, complicating patient handling and increasing risks during mobilization; and invasive intracranial monitoring, posing risks of device displacement or intracranial pressure instability (14–19). These factors collectively render conventional early mobilization particularly challenging or infeasible.

Given these constraints, passive head-up tilt positioning utilizing a tilt table has emerged as a viable alternative method for early mobilization. This technique addresses impaired consciousness by not requiring active patient cooperation, mitigates the impact of sedation and paralysis by passively supporting patients, reduces risks associated with mechanical ventilation by providing a stable and controlled posture, and accommodates invasive intracranial monitoring by minimizing the risk of device displacement or intracranial pressure fluctuations. Thus, passive verticalization potentially promotes circulation, decreases complications associated with prolonged immobilization, and supports neurological recovery through a feasible and controlled approach. While the efficacy of passive mobilization has been well-established in general ICU populations, its application and effectiveness in the neurocritical care environment have not been sufficiently investigated.

Therefore, this study aims to evaluate the clinical effectiveness of passive head-up tilt positioning using a tilt table in neurocritical patients, specifically examining impacts on intracranial pressure (ICP), delirium resolution, respiratory function, length of hospital stay, and overall patient outcomes.

## Materials and methods

### Study design and participants

This was a retrospective matched cohort study incorporating prospectively collected intervention data, conducted at the Department of Neurosurgery, Third Affiliated Hospital of Soochow University. We included critically ill patients admitted to the neurosurgical intensive care unit (NSICU) between July 2019 and June 2021 with a primary diagnosis of either traumatic brain injury or hypertensive intracerebral hemorrhage. During the intervention phase (July 2020 to June 2021), all patients who met the inclusion and exclusion criteria were consecutively enrolled in the verticalization group without any exclusion based on prognosis, family preference, or anticipated outcomes. The control group consisted of matched patients from the prior year (July 2019 to June 2020), before the verticalization protocol was introduced, ensuring temporal separation between groups and avoiding treatment crossover or contamination.

**Inclusion criteria** were: (1) age ≥ 45 years; (2) diagnosis of traumatic brain injury or hypertensive intracerebral hemorrhage; (3) stable vital signs; and (4) Glasgow Coma Scale (GCS) score between 3 and 9 on admission.

**Exclusion criteria** included: (1) terminal illness; (2) unstable vital signs (e.g., blood pressure, heart rate, respiratory rate, or oxygen saturation); (3) elevated intracranial pressure (ICP ≥ 15 mmHg); (4) Richmond Agitation-Sedation Scale (RASS) ≥ +4; (5) presence of deep vein thrombosis (DVT) in the lower extremities; (6) severe cardiac or pulmonary dysfunction; (7) multiple severe trauma; (8) active pneumothorax; (9) unstable fractures or spinal cord injury precluding mobilization; and (10) refusal or absence of informed consent from legal surrogates.

Between July 2020 and June 2021, 34 patients underwent passive verticalization. Of these, 5 were excluded due to incomplete physiological data (*n* = 2), loss to follow-up (*n* = 2), or early withdrawal from care based on family decision (*n* = 1). Thus, a total of 29 patients were included in the intervention group for final analysis.

### Intervention protocol

The passive verticalization intervention was conducted by a multidisciplinary team consisting of 1–2 neurosurgeons, 1–2 nurses, 1–2 nursing assistants, and family members. A motorized standing bed (model Carelead a-1) was used to implement the intervention.

Intervention procedure:

1. Ensure airway security, suspend intravenous infusions temporarily, and confirm ventilator connections.
2. Clamp external drainage tubes (e.g., ventricular, thoracic) if applicable. Maintain continuous electrocardiogram (ECG) and ICP monitoring.
3. Transfer the patient to the standing bed and secure them using safety straps.
4. Elevate the bed in three sequential stages: 30° for 7 min, 60° for 6 min, and 80° for 7 min. A 1-min supine rest period was included prior to returning the patient to the hospital bed.
5. Vital signs and ICP were continuously monitored throughout. The session was terminated if the patient developed systolic blood pressure >200 mmHg or <90 mmHg, marked diaphoresis, tachycardia, respiratory distress, SpO₂ < 80%, or ICP elevation >20 mmHg. This three-stage protocol (30° for 7 min, 60° for 6 min, and 80° for 7 min) was developed by adapting parameters from prior tilt-table mobilization studies in ICU and stroke rehabilitation settings (20–26). In the absence of a standardized neurocritical care protocol, our team refined this approach based on internal pilot experience, monitoring real-time changes in intracranial pressure, heart rate, and blood pressure. This stepwise strategy was designed to balance postural effectiveness and patient safety and was ultimately adopted as an institutional standard of care.

### Group allocation

Patients admitted between July 2020 and June 2021 who received passive verticalization and met the predefined inclusion/exclusion criteria were assigned to the intervention group. For the control group, matched patients were selected from the prior year (July 2019 to June 2020), before protocol implementation.

One-to-one matching was conducted based on five pre-specified variables:

1. age (±5 years),
2. sex,
3. primary diagnosis (traumatic brain injury or hypertensive intracerebral hemorrhage),
4. admission Glasgow Coma Scale (GCS) score (±1 point), and
5. APACHE II score (±3 points).

These variables were chosen due to their established influence on neurocritical outcomes. The matching process was performed manually using a pre-specified algorithm by an independent investigator who was blinded to all clinical outcomes, in order to minimize selection bias and enhance methodological rigor.

The final matched pairs showed no significant differences in baseline clinical or demographic characteristics (Table 1), indicating successful group comparability.

**Table 1:** Demographic and clinical characteristics of neurocritical patients at admission.

Item	Subcategory	Study group (*n* = 29)	Control group (*n* = 29)	*p*-value
Sex, n (%)	Male	18 (62.07)	19 (65.52)	0.785
	Female	11 (37.93)	10 (34.48)	
Age (years)	45–75	25 (86.21)	24 (82.76)	0.717
	>75	4 (13.79)	5 (17.24)	
Weight (kg)	-	65.48 ± 8.30	63.76 ± 7.83	0.419
Height (cm)	-	169.72 ± 5.40	169.76 ± 5.07	0.980
BMI (kg/m^2^)	-	22.65 ± 1.87	22.04 ± 1.67	0.197
Admission GCS	3–5	6 (20.69)	5 (17.24)	0.738
	6–9	23 (79.31)	24 (82.76)	
APACHE II score	-	27.07 ± 4.28	25.55 ± 4.43	0.190
Admission diagnosis, n (%)	TBI	17 (58.62)	15 (51.72)	0.597
	ICH	12 (41.38)	14 (48.28)	
Etiology, n (%)	Traffic accident	8 (27.59)	8 (27.59)	0.764
	Fall	6 (20.69)	6 (20.69)	
	High-altitude fall	3 (10.34)	1 (3.44)	
	Assault	0 (0.00)	0 (0.00)	
	Hypertension	12 (41.38)	14 (48.28)	
Lesion location, n (%)	Diffuse	2 (6.90)	3 (10.34)	0.850
	Right cerebral hemisphere	13 (44.83)	9 (31.03)	
	Left cerebral hemisphere	9 (31.03)	11 (37.93)	
	Bilateral cerebral hemispheres	2 (6.90)	3 (10.34)	
	Cerebellum	1 (3.45)	2 (6.90)	
	Brainstem	2 (6.90)	1 (3.45)	
Risk factors, n (%)	Hypertension	13 (44.83)	15 (51.72)	0.853
	Heart disease	2 (6.90)	3 (10.34)	
	Diabetes mellitus	3 (10.34)	4 (13.79)	
	Hematological disorder	1 (3.44)	0 (0.00)	
	Others	2 (6.90)	3 (10.34)	

TBI, Traumatic brain injury; ICH, Intracranial hemorrhage; GCS, Glasgow Coma Scale; APACHE, Acute Physiology and Chronic Health Evaluation. Continuous variables presented as mean ± SD, categorical variables as n (%).

### Data collection

Demographic and baseline clinical variables included age, sex, body mass index (BMI), admission GCS score, Acute Physiology and Chronic Health Evaluation II (APACHE II) score, primary diagnosis, etiology, lesion location, and comorbidities (e.g., hypertension, diabetes, cardiovascular disease, hematologic disorders).

### Clinical and neurological outcomes

Clinical and neurological outcomes were assessed using the following standardized scales: Glasgow Coma Scale (GCS), Coma Recovery Scale–Revised (CRS-R), Levels of Cognitive Functioning (LCF), Intensive Care Delirium Screening Checklist (ICDSC), Functional Independence Measure (FIM), Activities of Daily Living (ADL), Glasgow Outcome Scale (GOS), and Disability Rating Scale (DRS).

Assessments were conducted at multiple time points, including baseline (at admission), post-intervention (2 h after verticalization), discharge, 3-month follow-up, and 6-month follow-up. APACHE II was used to assess baseline illness severity. GCS, CRS-R, and LCF were assessed at baseline and discharge; DRS, FIM, and ADL were measured at 6 months post-discharge; GOS was recorded at 3 months. ICDSC was assessed before and after each verticalization session to evaluate delirium dynamics. Detailed scoring results and timelines are presented in the Results section.

### Physiological monitoring in the intervention group

Physiological parameters were systematically monitored in the intervention group to assess the safety and efficacy of passive verticalization. These included:

Intracranial dynamics: ICP, cerebral perfusion pressure (CPP), and pressure reactivity index (PRx)

Respiratory mechanics: static compliance (Cstat), inspiratory resistance (RI), and expiratory resistance (RE)

Neurological function: GCS, CRS-R, and ICDSC

Abdominal pressure: IAP.

All measurements were collected at standardized time points relative to each intervention session. Specifically, ICP, CPP, PRx, Cstat, RI, RE, GCS, CRS-R, and ICDSC were recorded at “Before” (2 h prior to verticalization) and “After” (2 h after verticalization). IAP was assessed at T0 (10 min before verticalization), T1 (10 min after initiation), and T2 (2 h post-intervention).

Neurological scores: GCS, CRS-R, and ICDSC before and after intervention.

Additional outcomes included NSICU length of stay (LOS), total hospital LOS, total hospitalization cost, intubation and mechanical ventilation duration, and incidence of complications (e.g., DVT and infections).

### Statistical analysis

All statistical analyses were performed using SPSS version 26.0 and GraphPad Prism. Continuous variables were expressed as mean ± standard deviation (SD) or median with interquartile range (IQR), and compared using Student’s t-test or Mann–Whitney U test based on distribution. Categorical variables were expressed as frequencies and percentages and compared using the chi-square test or Fisher’s exact test.

Multivariable logistic regression was conducted to assess the independent association between passive verticalization and infection outcomes, adjusting for potential confounders such as age, sex, APACHE II, and baseline neurological scores. A two-sided *p*-value <0.05 was considered statistically significant.

## Results

1. Baseline characteristics

A total of 58 patients were enrolled in the study, with 29 assigned to the intervention group and 29 to the control group. Three patients were lost to follow-up due to incorrect contact information. Baseline demographic and clinical characteristics, including age, sex, BMI, admission GCS and APACHE II scores, primary diagnosis, lesion location, and comorbidities, showed no statistically significant differences between groups (*p* > 0.05; Table 1).

2. Hospitalization metrics

**Figure 1:**
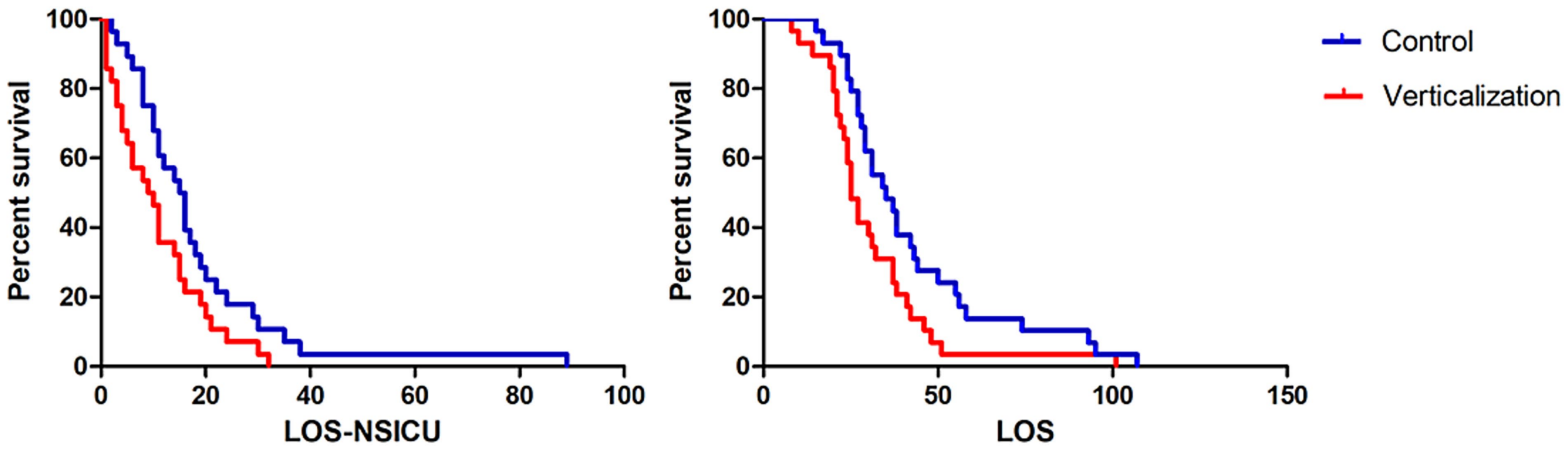
Comparison of NSICU and total hospital length of stay between groups. The intervention group receiving passive verticalization exhibited significantly shorter neurosurgical intensive care unit (NSICU) and total hospital length of stay compared to the control group (*p* < 0.05), indicating enhanced hospitalization efficiency associated with early mobilization.

Patients in the intervention group had significantly shorter NSICU length of stay (LOS) and overall hospital LOS compared to the control group (*p* < 0.05; Figure 1). Additionally, total hospitalization cost was lower in the intervention group (¥138,242.2 vs. ¥162,069.17), indicating improved cost-effectiveness.

3. Physiological monitoring during passive verticalization

Twenty-nine patients in the intervention group underwent standardized passive head-up tilt positioning, with each session lasting 20 min. Physiological parameters were collected at predefined intervals, as detailed in the Methods section.

**Figure 2:**
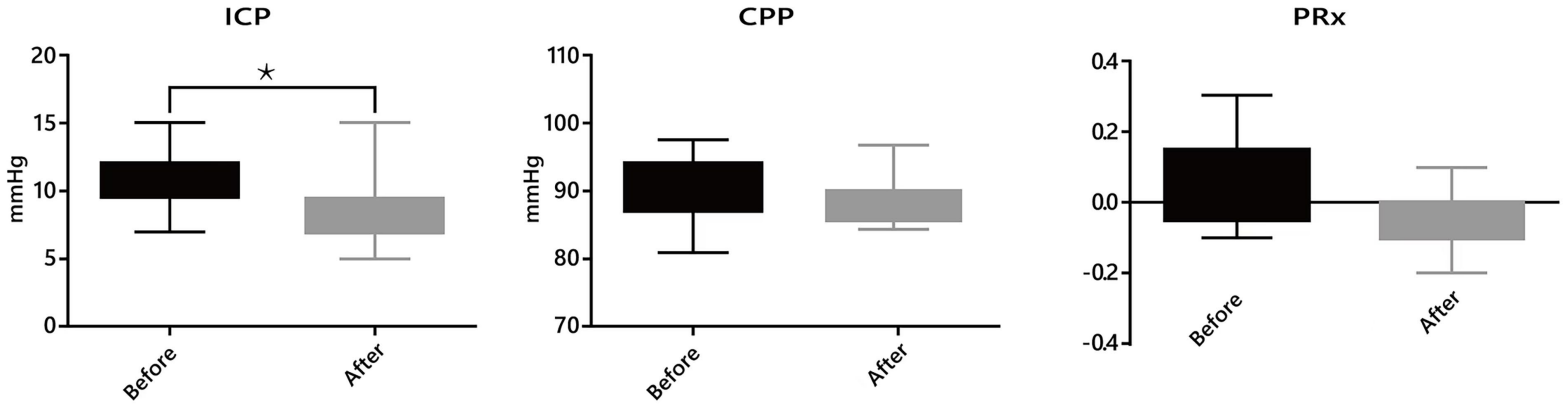
Changes in neurological and cognitive function following passive head-up tilt positioning. Scores on the Glasgow Coma Scale (GCS), Coma Recovery Scale-Revised (CRS-R), and Intensive Care Delirium Screening Checklist (ICDSC) were significantly improved after the intervention in the passive verticalization group (*p* < 0.05), indicating enhanced neurological recovery and reduced delirium severity.

**Figure 3:**
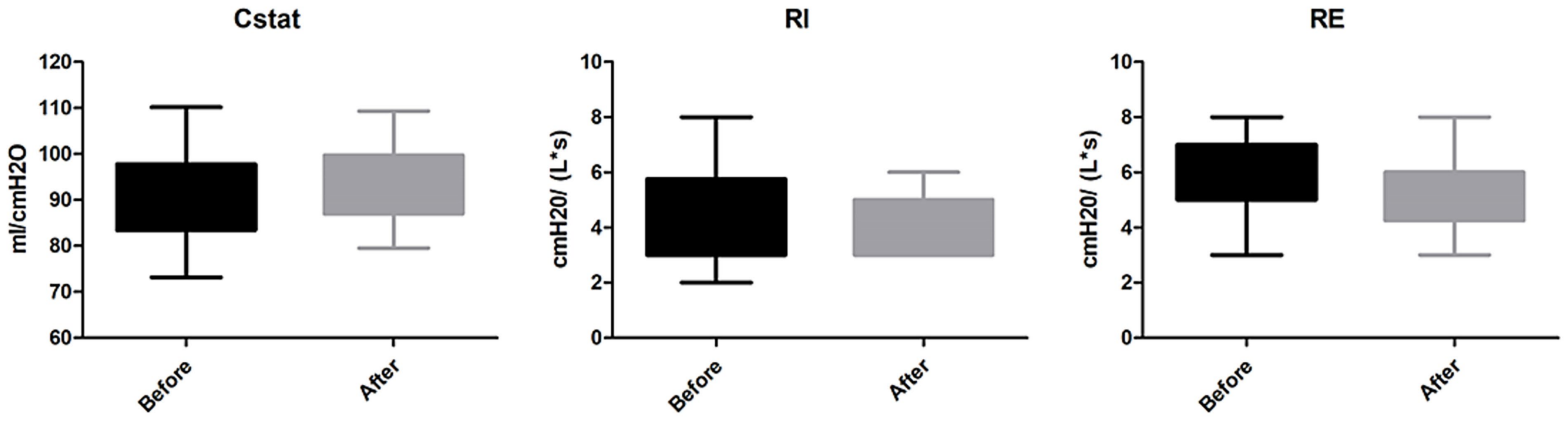
Effects of passive verticalization on intracranial dynamics. In patients with intracranial pressure (ICP) monitoring, a significant reduction in ICP was observed after passive verticalization (*p* < 0.05), while cerebral perfusion pressure (CPP) and pressure reactivity index (PRx) remained unchanged, suggesting maintained cerebral autoregulation.

**Figure 4:**
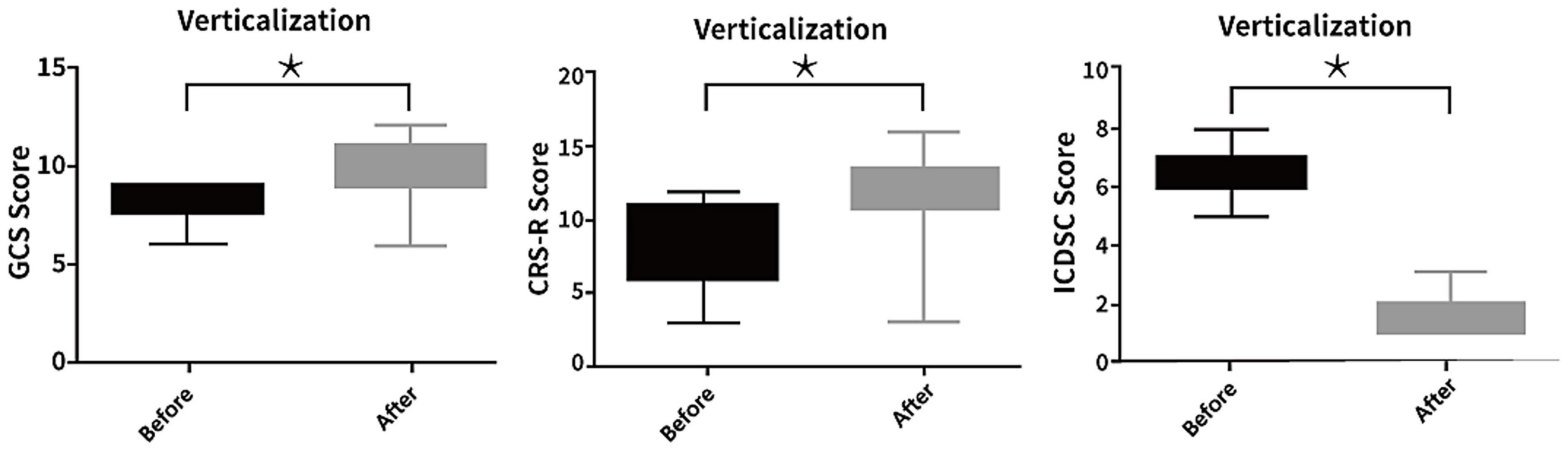
Impact of passive verticalization on mechanical ventilation and tracheostomy management. The intervention group exhibited a significantly shorter duration of mechanical ventilation and tracheostomy tube retention compared to controls (*p* < 0.05), indicating potential benefits of early passive mobilization for airway management in neurocritical care.

- Intracranial hydrodynamics: Among 21 patients with ICP monitoring, a significant reduction in mean ICP was observed after passive verticalization (10.62 ± 2.13 vs. 8.38 ± 2.27 mmHg, *p* < 0.05), while CPP and PRx showed no significant changes (*p* > 0.05; Figure 2).
- Respiratory mechanics: Among 16 patients receiving mechanical ventilation, no significant differences were noted in Cstat, RI, or RE before and after the intervention (all *p* > 0.05; Figure 3).
- Neurological status: Marked improvements were observed in GCS (7.90 ± 1.11 to 10.07 ± 1.49, *p* < 0.05) and CRS-R scores (8.17 ± 3.10 to 12.03 ± 2.54, *p* < 0.05). Among 27 patients previously diagnosed with delirium, all demonstrated improvement based on ICDSC scores (5.97 ± 1.94 to 1.62 ± 0.82, *p* < 0.05; Figure 4).
- Intra-abdominal pressure: IAP increased significantly at T1 (38.14 ± 3.73 mmHg) compared to T0 (9.52 ± 2.68 mmHg) and T2 (9.07 ± 2.62 mmHg; all *p* < 0.05), indicating a transient rise during verticalization that returned to baseline thereafter (Table 2).

4. Airway management

**Table 2:** Dynamic changes in IAP during passive orthostatic positioning.

Item	T0	T1	T2	*p*
IAP(29)	9.52 ± 2.68	38.14 ± 3.73	9.07 ± 2.62	
T0-T1				<0.001
T0-T2				0.514
T1-T2				<0.001

IAP,Intra-abdominal pressure; T0, Baseline measurement (10 min before verticalization); T1, Early-phase measurement (10 min after initiation); T2, Delayed-phase measurement (2 h post-intervention).

Tracheostomy incidence did not significantly differ between groups (12 vs. 15, *p* > 0.05). Mechanical ventilation duration was also significantly reduced (6.50 ± 5.53 vs. 12.71 ± 10.60 days, *p* < 0.05), while intubation rates and durations showed no statistical difference (Table 3).

**Table 3:** Comparative mechanical ventilation parameters and endotracheal tube duration between study cohorts.

Item	Study group	Control group	*p*
T (n)	12	15	0.597
ETT (n)	25	23	0.487
ETT Duration(d)	5.68 ± 3.79	8.22 ± 6.72	0.241
MV (n)	16	14	0.276
MV Duration(d)	6.50 ± 5.53	12.71 ± 10.60	0.048

T, Tracheostomy; ETT, Endotracheal tube; MV, Mechanical ventilation.

**Figure 5:**
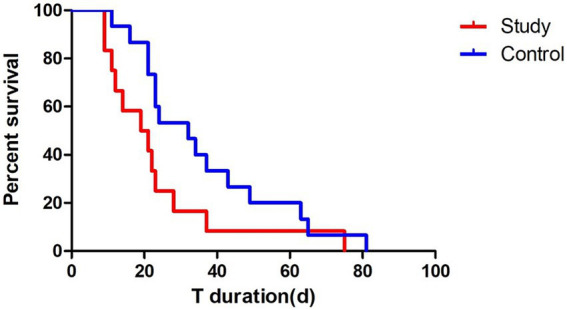
Kaplan–Meier survival analysis of tracheostomy tube retention. Survival analysis showed a significantly shorter tracheostomy tube retention time in the intervention group compared to the control group (log-rank test, *p* < 0.05), suggesting that passive verticalization may facilitate earlier decannulation.

To further assess the effect of passive verticalization on tracheostomy outcomes, Kaplan–Meier survival analysis was conducted using tracheostomy tube retention time as the event variable. (Figure 5). The intervention group demonstrated a significantly shorter tube duration compared to the control group (log-rank test, *p* < 0.05), indicating that passive head-up tilt positioning may facilitate earlier decannulation in neurocritical care patients.

5. Complications

The intervention group exhibited significantly shorter delirium duration (2.48 ± 0.58 vs. 4.08 ± 1.72 days, *p* < 0.05), earlier initiation of enteral nutrition (4.45 ± 1.24 vs. 5.59 ± 1.55 days, *p* < 0.05), and no cases of DVT (vs. 4 in the control group, *p* < 0.05). No significant differences were observed in the incidence of pulmonary infections, fever, or refractory hyperglycemia (*p* > 0.05; Table 4).

6. Prognosis and follow-up

**Table 4:** Comparative analysis of clinical complications between patient cohorts.

Item	Study group	Control group	*p*-value
Delirium (n)	27	24	0.227
Delirium Duration (d)	2.48 ± 0.58	4.08 ± 1.72	<0.001
Pulmonary Infection (n)	5	8	0.345
Fever Cases (n)	10	16	0.113
Fever Duration (d)	7.30 ± 3.59	9.13 ± 4.65	0.300
DVT (n)	0	4	0.038
EN Compliance Time (d)	4.45 ± 1.24	5.59 ± 1.55	0.004
IH (n)	2	5	0.227

DVT, Deep Vein Thrombosis; EN, Enteral Nutrition; IH, Intractable Hyperglycemia.

At discharge, patients in the intervention group showed significantly better GCS and LCF scores (*p* < 0.05). While 3-month follow-up revealed no significant differences in GOS, disability rate, or mortality, favorable trends were observed. At 6 months, ADL and DRS scores were significantly better in the intervention group (*p* < 0.05), suggesting enhanced long-term recovery (Table 5).

**Table 5:** Post-discharge prognosis and follow-up outcomes in the intervention and control groups.

Item	Study group	Control group	*p*
GCS	12.03 ± 2.82	9.83 ± 4.11	0.020
CRS-R	15.48 ± 6.17	12.59 ± 7.22	0.106
LCF	5.24 ± 2.31	3.86 ± 2.72	0.042
FIM	86.03 ± 36.27	71.31 ± 41.25	0.154
GOS (3 months)	3.55 ± 1.30	2.90 ± 1.40	0.066
3-month Disability	9(31.03)	13(44.83)	0.279
3-month Mortality	2(6.90)	5(17.24)	0.227
6-month ADL	66.21 ± 30.37	46.03 ± 36.73	0.026
6-month DRS	7.38 ± 7.85	12.38 ± 10.03	0.039

Scoring systems: GCS, Glasgow Coma Scale; CRS-R, Coma Recovery Scale–Revised; LCF, Levels of Cognitive Functioning; FIM, Functional Independence Measure; GOS, Glasgow Outcome Scale; ADL, Activities of Daily Living; DRS, Disability Rating Scale. The scoring ranges are as follows: GCS: 3 (deep coma or death) to 15 (fully alert); CRS-R: 0 (no response) to 23 (normal consciousness); LCF: 1 (no response) to 8 (purposeful and appropriate); FIM: 18 (total dependence) to 126 (complete independence); GOS: 1 (death) to 5 (good recovery); ADL: 0 (complete dependence) to 100 (fully independent); DRS: 0 (no disability) to 29 (extreme disability).

## Discussion

This study provides clinical evidence supporting passive head-up tilt positioning as an effective early mobilization strategy for patients in neurocritical care. The intervention was associated with reduced lengths of stay in both the NSICU and hospital overall, as well as significant improvements in neurological function and reduced incidence of common ICU-related complications. These findings align with previous evidence from general ICU populations, highlighting the value of early mobilization in enhancing recovery and preventing functional deterioration.

The observed improvement in GCS and CRS-R scores indicates that passive verticalization may facilitate arousal and neurological responsiveness. This may be attributed to increased cortical activation and enhanced sensory input during upright positioning, which has been proposed to stimulate the reticular activating system and promote consciousness (27, 28). More over, all patients diagnosed with delirium before intervention experienced marked improvements, as evidenced by substantial decreases in ICDSC scores. These findings align with prior research highlighting the role of early mobilization in mitigating delirium and enhancing cognitive function (29, 30).

A key finding of this study was the significant reduction in ICP following passive verticalization. Elevated ICP is a known risk factor for poor neurological outcomes, and effective, non-invasive interventions remain limited (31–36). The observed reduction is likely attributed to posture-induced modulation of CSF dynamics and enhanced cerebral venous drainage (20–23). Previous studies have shown that head elevation improves intracranial compliance and reduces ICP without adversely affecting CPP (24–26). Importantly, CPP and the PRx remained stable after verticalization, indicating preserved cerebral perfusion and autoregulatory function. This supports the safety of passive verticalization, even in patients with impaired consciousness or invasive monitoring. Unlike pharmacologic interventions, this approach is non-invasive, bedside-accessible, and can be safely implemented in early neurocritical care. Given its simplicity and physiological benefits, passive verticalization may serve as an adjunct to conventional ICP management. However, further studies are needed to define optimal positioning parameters and identify patients most likely to benefit from this intervention.

An intriguing physiological observation from our study was the transient increase in IAP during upright positioning, which returned to baseline upon resuming a supine position. This temporary elevation may stimulate gastrointestinal motility, potentially offering therapeutic benefits for neurogenic bowel dysfunction frequently encountered in neurocritical patients (37). Indeed, several patients reported relief from prolonged abdominal distension and improved bowel movements following intervention.

Additionally, regarding respiratory outcomes, the intervention significantly reduced the duration of mechanical ventilation and shortened tracheostomy tube retention. Although there was no difference in the number of patients requiring intubation or mechanical ventilation, shorter ventilation durations suggest that upright positioning may improve respiratory mechanics by optimizing lung volumes, reducing atelectasis, and enhancing diaphragmatic function, as supported by prior studies (38–42). These physiological improvements facilitate earlier restoration of airway protective reflexes, ultimately aiding earlier weaning from mechanical ventilation.

Importantly, a reduction in DVT incidence and faster attainment of enteral nutrition targets reflect the systemic benefits of early mobilization, even in patients unable to participate actively. These improvements may be due to enhanced venous return, improved hemodynamic stability, and reduced inflammatory response, all of which are known benefits of mobilization in critically ill patients (43, 44).

Long-term outcomes reinforced the benefits of passive verticalization, showing significantly better functional recovery as evidenced by improved GCS, Levels of LCF, ADL, and DRS scores at discharge and during follow-up evaluations. Although no statistically significant differences were observed in three-month disability and mortality rates, there was a consistent trend toward better outcomes in the intervention group. At 6 months post-discharge, the intervention group demonstrated superior functional independence and lower disability burden, highlighting the sustained impact of early passive mobilization on rehabilitation and quality of life.

This study provides novel clinical evidence supporting passive head-up positioning as a potentially effective adjunctive intervention in neurocritical care. Its integration into early rehabilitation protocols may contribute to improved neurological outcomes and reduced healthcare burden.

Nonetheless, this study has limitations, including a single-center, non-randomized design and relatively small sample size, which may affect generalizability and introduce selection bias. Future multicenter randomized controlled trials with larger cohorts are warranted to validate these findings and to establish standardized protocols for passive mobilization in neurocritical populations.

In addition, although we employed blinded manual matching and time-based group separation to minimize treatment selection bias, the inherent limitations of a retrospective design and non-randomized allocation remain. We acknowledge that future multicenter, prospective randomized trials are needed to validate these findings and confirm the safety and efficacy of passive verticalization under more controlled conditions.

## Conclusion

Passive head-up tilt positioning is a safe and effective early mobilization strategy for neurocritical care patients. This intervention was associated with reduced NSICU and hospital lengths of stay, shorter durations of mechanical ventilation and tracheostomy tube use, and improvements in neurological and cognitive recovery. It also lowered the incidence of complications such as delirium and DVT, contributing to better long-term functional outcomes. These findings highlight the potential of passive verticalization to support patients who are otherwise unsuitable for active mobilization due to impaired consciousness or invasive monitoring. By enhancing physiological stability and facilitating early functional recovery, this approach may improve care quality and resource utilization in neurocritical settings.

Further multicenter randomized controlled trials are needed to validate these findings and establish standardized protocols for passive verticalization in neurocritical care practice.

## Data Availability

The original contributions presented in the study are included in the article/supplementary material, further inquiries can be directed to the corresponding author.

## Glossary

NICU: neurointensive care unit
GCS: Glasgow Coma Scale
ICP: intracranial pressure
T0: 10 min before verticalization
T1: 10 min after initiation
T2: 2 h post-intervention
Before: 2 h prior to verticalization
After: 2 h after verticalization
CPP: cerebral perfusion pressure
PRx: pressure reactivity index
Cstat: static compliance
RI and RE: inspiratory and expiratory resistance
SD: standard deviation
LOS: lengths of stay
RASS: Richmond Agitation-Sedation Scale
DVT: deep vein thrombosis
ECG: electrocardiogram
BMI: body mass index
APACHE II: Acute Physiology and Chronic Health Evaluation II
CRS-R: Coma Recovery Scale-Revised
LCF: Cognitive Functioning
ICDSC: Intensive Care Delirium Screening Checklist
DRS: Disability Rating Scale
FIM: Functional Independence Measure
ADL: Activities of Daily Living
GOS: Glasgow Outcome Scale
IQR: interquartile range

## References

[1] Lang JK Paykel MS Haines KJ Hodgson CL. Clinical practice guidelines for early mobilization in the Icu: a systematic review. Crit Care Med. (2020) 48:e1121–8. doi: 10.1097/ccm.0000000000004574, PMID: 32947470

[2] Kho ME Connolly B. From strict bedrest to early mobilization: a history of physiotherapy in the intensive care unit. Crit Care Clin. (2023) 39:479–502. doi: 10.1016/j.ccc.2023.01.003, PMID: 37230552

[3] Hemphill JC 3rd Greenberg SM Anderson CS Becker K Bendok BR Cushman M. Guidelines for the Management of Spontaneous Intracerebral Hemorrhage: a guideline for healthcare professionals from the American Heart Association/American Stroke Association. Stroke. (2015) 46:2032–60. doi: 10.1161/str.0000000000000069, PMID: 26022637

[4] Hashem MD Nelliot A Needham DM. Early mobilization and rehabilitation in the Icu: moving Back to the future. Respir Care. (2016) 61:971–9. doi: 10.4187/respcare.04741, PMID: 27094396

[5] Zang K Chen B Wang M Chen D Hui L Guo S. The effect of early mobilization in critically ill patients: a Meta-analysis. Nurs Crit Care. (2020) 25:360–7. doi: 10.1111/nicc.12455, PMID: 31219229

[6] Rosa D Negro A Marcomini I Pendoni R Albabesi B Pennino G. The effects of early mobilization on acquired weakness in intensive care units: a literature review. Dimens Crit Care Nurs. (2023) 42:146–52. doi: 10.1097/dcc.0000000000000575, PMID: 36996359

[7] Alaparthi GK Gatty A Samuel SR Amaravadi SK. Effectiveness, safety, and barriers to early mobilization in the intensive care unit. Crit Care Res Pract. (2020) 2020:1–14. doi: 10.1155/2020/7840743, PMID: 33294221 PMC7714600

[8] Ruo Yu L Jia Jia W Meng Tian W Tian Cha H Ji Yong J. Optimal timing for early mobilization initiatives in intensive care unit patients: a systematic review and network meta-analysis. Intensive Crit Care Nurs. (2024) 82:103607. doi: 10.1016/j.iccn.2023.103607, PMID: 38158250

[9] Kumar MA Romero FG Dharaneeswaran K. Early mobilization in Neurocritical care patients. Curr Opin Crit Care. (2020) 26:147–54. doi: 10.1097/mcc.0000000000000709, PMID: 32068582

[10] Klein K Mulkey M Bena JF Albert NM. Clinical and psychological effects of early mobilization in patients treated in a neurologic Icu: a comparative study. Crit Care Med. (2015) 43:865–73. doi: 10.1097/ccm.0000000000000787, PMID: 25517476

[11] Titsworth WL Hester J Correia T Reed R Guin P Archibald L. The effect of increased mobility on morbidity in the Neurointensive care unit. J Neurosurg. (2012) 116:1379–88. doi: 10.3171/2012.2.Jns111881, PMID: 22462507

[12] Rand ML Darbinian JA. Effect of an evidence-based mobility intervention on the level of function in acute intracerebral and subarachnoid hemorrhagic stroke patients on a Neurointensive care unit. Arch Phys Med Rehabil. (2015) 96:1191–9. doi: 10.1016/j.apmr.2015.02.008, PMID: 25701637

[13] Witcher R Stoerger L Dzierba AL Silverstein A Rosengart A Brodie D. Effect of early mobilization on sedation practices in the neurosciences intensive care unit: a Preimplementation and Postimplementation evaluation. J Crit Care. (2015) 30:344–7. doi: 10.1016/j.jcrc.2014.12.003, PMID: 25573283

[14] Bauerschmidt A Al-Bermani T Ali S Bass B Dorilio J Rosenberg J. Modern sedation and analgesia strategies in Neurocritical care. Curr Neurol Neurosci Rep. (2023) 23:149–58. doi: 10.1007/s11910-023-01261-7, PMID: 36881257

[15] Prabhakar H Tripathy S Gupta N Singhal V Mahajan C Kapoor I. Consensus statement on Analgo-sedation in Neurocritical care and review of literature. Indian J Crit Care Med. (2021) 25:126–33. doi: 10.5005/jp-journals-10071-23712, PMID: 33707888 PMC7922463

[16] Ziaka M Exadaktylos A. Brain-lung interactions and mechanical ventilation in patients with isolated brain injury. Crit Care. (2021) 25:358. doi: 10.1186/s13054-021-03778-0, PMID: 34645485 PMC8512596

[17] Yang R Eagles ME. Methods of monitoring intracranial pressure: a review. Neurosurg Clin N Am. (2025) 36:141–7. doi: 10.1016/j.nec.2024.11.003, PMID: 40054968

[18] Provencio JJ. Neurocritical care research: collaborations for curing coma. Crit Care Clin. (2023) 39:47–54. doi: 10.1016/j.ccc.2022.08.001, PMID: 36333036

[19] Oberholzer M Müri RM. Neurorehabilitation of traumatic brain injury (TBI): a clinical review. Med Sci (Basel). (2019) 7:47. doi: 10.3390/medsci7030047, PMID: 30889900 PMC6473767

[20] Lachance BB Chang W Motta M Parikh G Podell J Badjatia N. Verticalization for refractory intracranial hypertension: a case series. Neurocrit Care. (2022) 36:463–70. doi: 10.1007/s12028-021-01323-z, PMID: 34405321

[21] Wolfe TJ Torbey MT. Management of Intracranial Pressure. Curr Neurol Neurosci Rep. (2009) 9:477–85. doi: 10.1007/s11910-009-0070-1 19818235

[22] Johnson JN Teton ZE Lee JE Bergsneider M Srinivasan VM Colby GP. The human Craniospinal venous system and its influence on postural intracranial pressure: a review. J Neurosurg. (2024) 141:1484–93. doi: 10.3171/2024.4.Jns232254, PMID: 39029117

[23] Barami K Sood S. The cerebral venous system and the postural regulation of intracranial pressure: implications in the Management of Patients with cerebrospinal fluid diversion. Childs Nerv Syst. (2016) 32:599–607. doi: 10.1007/s00381-015-3010-1, PMID: 26767844

[24] Ramos MB Britz JPE Telles JPM Nager GB Cenci GI Rynkowski CB. The effects of head elevation on intracranial pressure, cerebral perfusion pressure, and cerebral oxygenation among patients with acute brain injury: a systematic review and Meta-analysis. Neurocrit Care. (2024) 41:950–62. doi: 10.1007/s12028-024-02020-3, PMID: 38886326

[25] Che Y Lu T Wang T Zhao H Song X Zhan Q. A Meta-analysis of the clinical efficacy of the head-of-bed elevation for patients with acquired brain injury. J Neurosci Nurs. (2023) 55:91–6. doi: 10.1097/jnn.0000000000000703, PMID: 37094377

[26] Ng I Lim J Wong HB. Effects of head posture on cerebral hemodynamics: its influences on intracranial pressure, cerebral perfusion pressure, and cerebral oxygenation. Neurosurgery. (2004) 54:593–8. doi: 10.1227/01.neu.0000108639.16783.39, PMID: 15028132

[27] Nudo RJ. Neural bases of recovery after brain injury. J Commun Disord. (2011) 44:515–20. doi: 10.1016/j.jcomdis.2011.04.004, PMID: 21600588 PMC3162095

[28] Bonanno M De Luca R De Nunzio AM Quartarone A Calabrò RS. Innovative technologies in the neurorehabilitation of traumatic brain injury: a systematic review. Brain Sci. (2022) 12:1678. doi: 10.3390/brainsci12121678, PMID: 36552138 PMC9775990

[29] Nydahl P Jeitziner MM Vater V Sivarajah S Howroyd F McWilliams D. Early mobilisation for prevention and treatment of delirium in critically ill patients: systematic review and Meta-analysis. Intensive Crit Care Nurs. (2023) 74:103334. doi: 10.1016/j.iccn.2022.103334, PMID: 37440187

[30] Sadlonova M von Arnim CAF. Update on the diagnosis and treatment of delirium. Inn Med (Heidelb). (2023) 64:855–63. doi: 10.1007/s00108-023-01561-7, PMID: 37540259

[31] Yue JK Rick JW Deng H Feldman MJ Winkler EA. Efficacy of decompressive Craniectomy in the Management of Intracranial Pressure in severe traumatic brain injury. J Neurosurg Sci. (2019) 63:425–40. doi: 10.23736/s0390-5616.17.04133-9, PMID: 29115100

[32] Kareemi H Pratte M English S Hendin A. Initial diagnosis and Management of Acutely Elevated Intracranial Pressure. J Intensive Care Med. (2023) 38:643–50. doi: 10.1177/08850666231156589, PMID: 36802976 PMC10302390

[33] Zoerle T Beqiri E Åkerlund CAI Gao G Heldt T Hawryluk GWJ. Intracranial pressure monitoring in adult patients with traumatic brain injury: challenges and innovations. Lancet Neurol. (2024) 23:938–50. doi: 10.1016/s1474-4422(24)00235-7, PMID: 39152029

[34] Changa AR Czeisler BM Lord AS. Management of Elevated Intracranial Pressure: a review. Curr Neurol Neurosci Rep. (2019) 19:99. doi: 10.1007/s11910-019-1010-3, PMID: 31773291

[35] Cook AM Morgan Jones G Hawryluk GWJ Mailloux P McLaughlin D Papangelou A. Guidelines for the acute treatment of cerebral edema in Neurocritical care patients. Neurocrit Care. (2020) 32:647–66. doi: 10.1007/s12028-020-00959-7, PMID: 32227294 PMC7272487

[36] Schizodimos T Soulountsi V Iasonidou C Kapravelos N. An overview of Management of Intracranial Hypertension in the intensive care unit. J Anesth. (2020) 34:741–57. doi: 10.1007/s00540-020-02795-7, PMID: 32440802 PMC7241587

[37] Rivi E Filippi M Fornasari E Mascia MT Ferrari A Costi S. Effectiveness of standing frame on constipation in children with cerebral palsy: a single-subject study. Occup Ther Int. (2014) 21:115–23. doi: 10.1002/oti.1370, PMID: 24838311

[38] Yamada Y Yamada M Chubachi S Yokoyama Y Matsuoka S Tanabe A. Comparison of inspiratory and expiratory lung and lobe volumes among supine, standing, and sitting positions using conventional and upright Ct. Sci Rep. (2020) 10:16203. doi: 10.1038/s41598-020-73240-8, PMID: 33004894 PMC7530723

[39] Lai CC Chou W Chan KS Cheng KC Yuan KS Chao CM. Early mobilization reduces duration of mechanical ventilation and intensive care unit stay in patients with acute respiratory failure. Arch Phys Med Rehabil. (2017) 98:931–9. doi: 10.1016/j.apmr.2016.11.007, PMID: 27979608

[40] Zhang L Hu W Cai Z Liu J Wu J Deng Y. Early mobilization of critically ill patients in the intensive care unit: a systematic review and Meta-analysis. PLoS One. (2019) 14:e0223185. doi: 10.1371/journal.pone.0223185, PMID: 31581205 PMC6776357

[41] Worraphan S Thammata A Chittawatanarat K Saokaew S Kengkla K Prasannarong M. Effects of inspiratory muscle training and early mobilization on weaning of mechanical ventilation: a systematic review and network Meta-analysis. Arch Phys Med Rehabil. (2020) 101:2002–14. doi: 10.1016/j.apmr.2020.07.004, PMID: 32750371

[42] Zafiropoulos B Alison JA McCarren B. Physiological responses to the early mobilisation of the intubated, ventilated abdominal surgery patient. Aust J Physiother. (2004) 50:95–100. doi: 10.1016/s0004-9514(14)60101-x, PMID: 15151493

[43] Li Q Yu Z Chen X Wang J Jiang G. Risk factors for deep venous thrombosis of lower limbs in postoperative neurosurgical patients. Pak J Med Sci. (2016) 32:1107–10. doi: 10.12669/pjms.325.10481, PMID: 27882003 PMC5103115

[44] Lei YT Xie JW Huang Q Huang W Pei FX. Benefits of early ambulation within 24 H after Total knee arthroplasty: a multicenter retrospective cohort study in China. Mil Med Res. (2021) 8:17. doi: 10.1186/s40779-021-00310-x, PMID: 33673879 PMC7934453

